# Stance markers in English medical research articles and newspaper opinion columns: A comparative corpus-based study

**DOI:** 10.1371/journal.pone.0247981

**Published:** 2021-03-08

**Authors:** Qian Shen, Yating Tao

**Affiliations:** English Department, North China Electric Power University, Baoding, Hebei, China; STL UMR8163 CNRS, FRANCE

## Abstract

Stance markers are critical linguistic devices for writers to convey their personal attitudes, judgments or assessments about the proposition of certain messages. Following Hyland’s framework of stance, this study investigated the distribution of stance markers in two different genres: medical research articles (medical RA) and newspaper opinion columns (newspaper OC). The corpus constructed for the investigation includes 52 medical research articles and 175 newspaper opinion articles, which were both written in English and published from January to April in 2020 with the topic focusing on COVID-19. The findings of this study demonstrated that the occurrences of stance markers in newspaper OC were far more frequent than those in medical RA, indicating the different conventions of these two genres. Despite the significant difference in the occurrences of stance markers between the two sub-corpora, similarities of the most frequent stance markers in two genres were also highlighted. The study indicated that the topic content seems to play an important role in shaping the way of how writers construct their stance. The lack of information or evidence on the topic of COVID-19 could restrain writers from making high degree of commitment to their claims, which make them adopt a more tentative stance to qualify their statements.

## 1. Introduction

Throughout the last four decades, stance has received much attention from linguists with various approaches applied to its study. The concept of stance proposed by Biber [1] is to convey a speaker or writer’s personal attitudes, feelings, judgments or assessments about the proposition of certain messages. This linguistic mechanism is also studied under various paradigms with their own focuses and approaches, including *evidentiality* [2], *affect* [3], *attitude* [4], *evaluation* [5], *appraisal* [6] and *metadiscourse* [7]. Those approaches attempt to reveal how writers project themselves into their discourse and signal their attitudes towards the propositions presented in the discourse. They all have drawn on a distinction between meanings that express the writer’s positioning with respect to their commitment to the reliability of the proposition, and those that indicate the writer’s personal feelings and attitudes towards the proposition. No matter which superordinate term of expressing attitudes is preferred by scholars, their common interests are to explore how various linguistic devices reflect authors’ beliefs and values and help authors establish effective interpersonal relationship with their readers.

Stance can be realized through grammatical devices and lexical words, which express epistemic knowledge (e.g. *might*, *suggest*, *probably*, *possibility*, *likely*) and authors’ attitudes towards propositions (e.g. *unfortunately*, *surprisingly*) [8]. Considerable research has been conducted to discover how writers use different linguistic resources to persuade readers and convince the discourse community with their claims [9–20]. Some prior work has detailed the presence of stance markers across academic registers, e.g. graduate student theses [11], a range of spoken and written academic registers from textbooks to lectures [12], and undergraduate writing [13]. One particular productive area of stance research has examined stance features in different academic research articles (RA), e.g. hedging in RA of molecular biology [14, 15], stance markers in results sections of experimental RA by biochemists [16], construction of stance in reporting clauses from a cross-disciplinary perspective [11], stance and engagement in pure mathematics research articles [17], stance in dissertation acknowledgements [18], stance-taking in discussion sections of applied linguistics RA [19] and stance construction in biochemical RA [20].

In addition to the above-mentioned studies, there is also some research dealing with stance construction in newspaper opinion articles and popular science. Hyland [21] examined a corpus of texts in research papers and popular science articles, and shed some light on the ways of how writers manage their expertise and interactions with readers through rhetorical resources. Fu & Hyland [22] drew on Hyland’s model of interactional metadiscourse to explore how different linguistic devices were employed in popular science and opinion articles. However, little corpus-based comparative research has been conducted on the distributional similarities and differences of stance markers between RA and opinion columns (OC). The present study seeks to extend the understanding of stance features by exploring rhetorical choices used to express writers’ stance towards the same topic in two different genres: medical RA and newspaper OC.

### 1.1 Research on medical research writing

Previous studies in medical research writing focused predominantly on the analysis of rhetorical moves in medical RA [23–29]. Nwogu [24] examined the whole body of thirty medical RA from five journals and his findings revealed an eleven-move schema with eight obligatory and three optional moves. Similarly, Li & Ge [25] adopted a diachronic approach to identify the moves in fifty medical RA published between two different periods in the same journals. Based on a combined methodology, Fryer [26] examined the generic rhetorical moves of sixteen experimental medical RA dealing with certain topic through a systemic-functional approach. In addition to describing all the moves in medical RA, many researchers have also looked at rhetorical moves in individual sections of medical RA, e.g. results sections of four clinical reports [27], function analysis of discussion sections of medical RA from the *British Medical Journal* [28], methods sections of medical RA from the *British Medical Journal* [29].

Another area of investigation into medical RA is concerned with particularly linguistic features of medical written discourse. Thomas & Hawes [30] identified three major categories of verbs used in reporting statements in medical RA and described their roles in the discourse. Their findings suggested there is a correlation between the choice of verb type and the different functions realized by these verbs. Salager-Meyer [31] and Varttala [32] both discussed the communicative function of hedges in different types of medical written discourse. Their selection of hedge expressions were based on different classifications, but their findings both highlighted that lexical hedging devices are remarkable resources for medical writing. Additionally, other linguistics features of medical RA were also investigated in the previous studies, such as self-reference [33], collocational frameworks [34] and metaphors in medical written discourse [35].

The above mentioned studies are mainly focused on rhetorical moves and linguistic features of medical RA, and there are a few studies which are also centred on the construction of stance in medical RA. Gray et al. [36] investigated the explicit expression of specific attitudes or assessments of epistemic status in medical prose from mid-seventeenth century to the early eighteenth century with the purpose of contrasting stance features in traditional medical texts with those in *Philosophical Transactions* from the same period. Their analysis focused on stance adverbials, modal and semi-modal verbs and *that*-complement clauses to reveal authors’ evaluations and commitment towards propositions conveyed in the articles. Yang et al. [37] explored the use of epistemic modality in English-medium medical RA, and their results indicated that medical RA written by native English-speaking writers are more likely to use tentative and objective presentation of their claims to construct new knowledge with the purpose of avoiding subjectivization of their statements. A more recent study conducted by Poole et al. [20] analyzed epistemic stance features in a diachronic corpus of biochemical research. Their results revealed an increase in the use of boosters and a decrease in the use of modal verbs over five time periods. Although those studies investigated certain aspects of stance construction such as grammatical constructions and epistemic modality, they did not go further to make a thorough investigation of different types of stance markers in medical RA. A more comprehensive study of stance markers in medical RA is needed in order to show how various linguistic devices are employed by medical research writers in their construction of stance and communication with readers.

### 1.2 Research on newspaper opinion column writing

The rationale behind choosing newspaper OC as one of the source data in this study is because they are persuasive in nature. Unlike newspaper editorials which are generally institutional [38], newspaper OC seek to provide readers a reliable “voice” and enhance readers’ knowledge and beliefs. The function of opinion discourse is to speak with a convincing “voice” to readers directly. The distinctive features of newspaper OC are that writers can explicitly manifest their subjective attitudes and open judgments of issues [39]. This characteristic demands conscious structuring of texts to create a bond between writers and readers.

Several investigations of newspaper opinion discourse have focused on the stance-taking in national newspapers. Marin-Arrese [40] explored the expressions of the author’s commitment to the validity of the information and the degree of subjectivity in two genres of newspaper discourse: opinion columns and leading articles. The findings of her study indicated there is no significant difference between the two genres in terms of the author’s commitment, whereas the two genres differ significantly in the dimension of subjectivity. Tavassoli et al. [41] analyzed the attitudinal language of articles and studied the stances towards the Syrian refugee in two British newspapers with different political orientations, *The Guardian* and *The Telegraph*. Their findings suggested that the attitudes adopted by newspapers have great potential to shape readers’ positioning on the refugees issue, either to show the welcoming stance or the unwelcoming stance.

Additionally, there are also some cross-linguistic investigations on the construction of stance in newspaper opinion discourses. Through a corpus-based contrastive study, Pérez-Blanco [42] analyzed negative evaluative adjectives in English and Spanish opinion discourse and discussed the observed cross-cultural differences in the construction of attitudinal stance. Drawing on Hyland’s model of stance markers, Babapour & Kuhi [43] conducted a contrastive study on English and Farsi newspaper OC to investigate the frequency of different types of stance markers. Their findings revealed that hedges and self-mentions used by English columnists are significantly more frequent than Farsi columnists, while Farsi columnists use a large number of boosters and attitude markers to construct their stance. These contrastive studies reveal interesting similarities and differences of stance expressions in different languages.

Unlike medical RA written for academic professionals and fellow researchers, opinion texts are usually written for non-specialist readers and seek to inform and convince a mass audience of non-experts of particular claims about the world. Considering the different types of readers, writers of different genres usually employ various rhetorical choices to persuade readers to engage in the discussion and align them with the writer’s perspective on the topic. Medical research writers wish to persuade the discourse community to accept that their specific experiment results are credible and plausible, whereas opinion columnists seek to contribute to wider debates concerning events in the world and shape readers’ attitudes towards the events. Although medical RA and newspaper OC belong to the different aspects of the publishing landscape, the comparative study of different genres focusing on the same topic will certainly highlight the different linguistic choices writers adopt to display their expertise, and at the same time show that interpersonal relationships can be established by telling readers what writers believe as important. Therefore, our comparative study of rhetorical resources in medical RA and newspaper OC concerning the topic of COVID-19 might help shed some light on the construction of stance and discourse practices employed in each of the two genres. Drawing on Hyland’s stance model [44], we hope to identify the typical stance features of these two genres with the purpose of addressing the following two questions:

1. What are the frequencies of stance markers used in medical RA and newspaper OC concerning the same topic?
2. What are the similarities and differences concerning the frequencies and typical features of the most frequently used stance markers in each of the two genres?

## 2. Materials and methods

### 2.1 Corpus description

Over the past three decades, the introduction of electronic language corpora has changed the way linguistic research is conducted. Real language data which is electronically stored and retrieved for research purpose provide new insights on language patterns [45]. For the purpose of this study, the two comparable sub-corpora of medical RA and newspaper OC were compiled to investigate similarities and differences of stance construction in these two genres.

The investigation of stance construction from a contrastive perspective is due to the following two aspects. First, the selection of data in both sub-corpora is based on the topic of COVID-19, which was not only widely reported in the news media but also vigorously investigated throughout the medical research fields especially during the early stage of COVID-19 outbreak. Second, there is a widely held belief that the persuasive characteristics of newspaper OC demands columnists’ conscious structuring of discourse to create interpersonal bonds between them and readers. The COVID-19 pandemic has brought uncertainty to people’s daily lives, and newspaper columnists show their attitudes towards the pandemic and make their concerns explicit to the public. This may demonstrate the pervasive use of stance markers in newspaper opinion discourse. In addition to the newspaper coverage of COVID-19, a number of medical studies were conducted on the causes, transmission patterns, syndromes and possible treatments of COVID-19 at the initial stage of outbreak. Besides medical RA, medical journals also publish reports, editorials, news and discussions on the topic of COVID-19. The reason that we chose medical RA instead of other article types is because medical research articles are original and empirical in nature, and the exploration of stance markers in medical RA might shed some light on how medical RA writers construct their attitudes towards research findings on COVID-19. Furthermore, language features and communicative purposes vary differently between different genres. Through the comparison between newspaper OC and medical RA, we want to find out how linguistic resources are employed in different genres to reflect author’s stance on the same topic.

To compile a corpus of medical RA, we first checked the impact factors of the most influential medical journals on line, and then compiled a potential list of journals with high impact factors in medical fields. Based on the publication countries, we finally chose the following seven medical journals: *The Lancet*, *Science*, *Nature*, *The British Medical Journal (BMJ)*, *The New England Journal of Medicine (NEJM)*, *Proceedings of the National Academy of Sciences of the United States of America (Pans)* and *The journal of the America Medical Association (JAMA)*. After identifying each medical journal, we went to the official website of each journal and used COVID-19 as the keyword to search for medical RA. In order to make our data homogeneous, we precluded other types of journal articles such as reports, discussion, and correspondence. Considering the development trend in the COVID-19 pandemic, we limited the online publication date of each article to a certain period from January to April in 2020, and finally selected 52 English-medium medical RA from those seven medical journals. After the collection of those articles, we manually deleted author titles, tables, graphs, acknowledgements and references in each article and converted them into an electronic corpus of 187, 043 tokens (see Table 1).

**Table 1:** Corpus composition.

Corpus	Tokens	Numbers of Texts
Medical Research Articles	187, 043 tokens	52
Newspaper Opinion Columns	189, 270 tokens	175

The newspaper corpus in this study was taken from the opinion column genre. We used a computer program code to help us collect articles concerning the topic of COVID-19 from opinion sections in *The New York Times*, which has developed a national and international reputation for thoroughness over time and has the most comprehensive coverage of reports in the world. We retrieved a total number of 375 opinion texts published from January to early April in 2020. In order to balance the size of medical corpus and newspaper corpus, 175 opinion texts were randomly selected to build the newspaper opinion corpus of 189, 270 tokens (see Table 1). Additionally, the keyword list of the newspaper corpus was also examined by corpus software to check whether the contents of opinion texts are related to COVID-19. The keyword list of the whole 175 opinion texts was examined through the comparison with the reference corpus of the Lancaster-Oslo/Bergen corpus, and we found that the top ten keywords in OC corpus include *coronavirus*, *covid*, *pandemic*, *virus*, *Trump*, *health*, *people*, *workers*, *crisis* and *we*. Those keywords are in high consistency with the topic of COVID-19, which further ensures the comparability of the data in medical RA and newspaper OC.

### 2.2 Analytic framework

**Fig 1:**
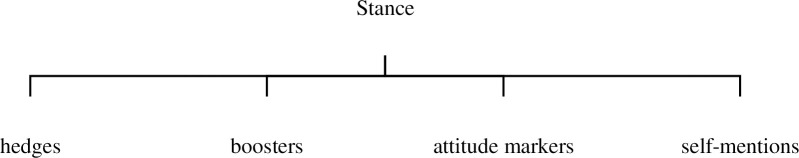
Hyland’s stance model.

This study primarily adopts the stance framework by Hyland [44]. The extensive research in the area of stance conducted by Hyland is closely connected with his early research on hedging and boosting in academic writing [14, 15, 46]. Hyland’s paradigm of stance is based on his interpersonal model of metadiscourse, in which stance is described as “writer-oriented features of interaction” and refers to the extent of the commitments they express to the credibility of a claim, or the attitude they convey to the contents of propositions [7]. Hyland’s stance framework is comprised of four elements: hedges, boosters, attitude markers and self-mentions (see Fig 1).

Hedges and boosters refer to the writer’s commitment to the credibility of the propositions [44]. Writers use hedge devices such as *might*, *possible* and *could* to withhold their commitment to a proposition, and calculate the degree of precision and reliability they would like their claims or assertions to carry. By means of hedges, information is thus usually presented as opinions rather than facts. Boosters, on the other hand, allow writers to use expressions such as *clearly*, *demonstrate* and *obvious* to convey certainty and show total commitment to the arguments they have made. Those boosting devices thus facilitate writers to present their claims with great assurance and also establish strong interpersonal solidarity with readers. Attitude markers reflect the writer’s personal feelings or assessments towards what is presented [44]. They enable writers to convey their affective meanings such as surprise, agreement, importance, frustration rather than their commitment to propositions. Attitude can be explicitly realized through the use of attitude verbs like *agree*, sentence adverbs like *fortunately*, and adjectives like *extraordinary*. Self-mention denotes the degree to which the writer wants to manifest him/herself in the text through first person pronouns and possessive adjectives [47]. It is unavoidable for writers to project their impressions on their arguments, their discipline and their readers [44]. The employment of explicit self-mention markers is a conscious choice to show writers’ authorial identity and their particular stance.

### 2.3 Analytical procedures

According to Biber & Jones [48], there are three major types of research design employed in corpus research. The major differences among these research designs lie in the unit of analysis: type A design focuses on the variants of a linguistics structure; type B studies the differences among texts and text varieties; type C design treats the entire corpus or different sub-corpora as the unit of analysis. Instead of treating each text as an observation, the present research belongs to the type C design, in which each sub-corpus is treated as a single observation. The rate of occurrence of linguistic features in each sub-corpus is computed and compared across different genres.

Based on Hyland’s [44] classification model, Biber [1], Hyland [10, 49], Holmes [50] and Peacock [51] were consulted in order to generate a composite list of stance markers. The list comprised 450 search items compiled from those previous studies on different genres and registers, including 154 hedging devices, 215 boosters, 66 attitude markers and 15 self-mentions. We divided the lists of words and phrases of each category into sub-categories of stance expressions (see Table 2) in order to capture fine-grained distinctions between the two sub-corpora. We then used the concordancing program AntConc [52] to conduct targeted searches of the four stance categories by examining the frequency of each word or phrase compiled in our list. We would like to point out that all those 450 linguistic items were not evenly distributed across the two sub-corpora. Some linguistic forms were more frequently used in one sub-corpus than the other, and some stance markers compiled in our list were not employed in either of the sub-corpora. After we completed this quantitative collection of potential candidates, we then moved on to the qualitative analysis through concordancing each example. Since stance markers are extremely varied and many linguistic devices are multifunctional, all instances of each stance marker were manually examined to verify that each item worked in the target function capacity.

**Table 2:** Stance framework in this study.

**Basic Elements**	**Sub-categorizations**	**Examples**
Hedges	Modal verbs	could, can, might, may
	Epistemic lexical verbs	suggest, indicate
	Adverbs, adjectives and nouns	probably, perhaps, likely, possibility
	Approximators	always, somewhat, about
	Other phrases	in this view, open to question
Boosters	Modal verbs	will, must
	Epistemic lexical verbs	show, demonstrate
	Adverbs and adjectives	certainly, important
	Attributors	As a former exam marker reveals. . .
	Others phrases	It is well-known that.. We can be sure that ..
Attitude markers	Deontic verbs	must, should
	Attitudinal adverbs	unfortunately, happily
	Attitude adjectives	interesting, absurd
	Cognitive verbs	I feel…, I believe….
Self-mentions	First person pronouns	I, me, we, us
	Nouns phrases	the writer, the author

To ensure the accuracy of the study, each researcher used AntConc to conduct the quantitative search of the targeted items and then manually checked each concordance line to identify whether the targeted item serve the interpersonal function of stance. All instances were cross-checked in original contexts by both authors independently, and the reliability index for inter-rater agreement of Cohen’s Kappa was over 0.8. When disagreements emerged from some fuzzy words like modal verbs, we resorted to both the contexts and Hoey’s [53] interpretation of the modal verbs to make sure the epistemic meaning would be appropriately assigned. For each result, the frequencies of occurrence of the items were normalized to occurrences per 1000 words. At the same vein, in order to figure out whether there are any distribution similarity or difference of stance markers in both medical RA and newspaper OC, chi-square test was employed to see if the differences are statistically significant.

## 3. Results

### 3.1 Macro-categorical distribution of stance markers in newspaper OC and medical RA

After a cautious and meticulous quantitative analysis, the results disclosed stance markers were frequently used in both genres, which corroborated that stance markers are important linguistic resources to achieve interaction in a text. The total occurrences of stance markers in newspaper OC were nearly twice as many as those in medical RA (see Table 3), reflecting a high level of interaction between writers and readers in newspaper OC. For example, self-mentions and attitude markers in newspaper OC (12.5 per 1000 words and 6.3 per 1000 words respectively) were far more frequent compared with those (8.0 per 1000 words and 2.2 per 1000 words respectively) in medical RA. In addition to counting the occurrences of each category, a chi-square test was also calculated to further analyze whether there were any statistical differences between OC and RA in terms of the frequencies. The results revealed statistically significant differences existed in four macro-categories between the two genres. Compared with medical research writers, newspaper columnists tend to use far more hedges (χ^2^ = 255.56, p<0.0001), boosters (χ^2^ = 408.76, p<0.0001), attitude markers (χ^2^ = 376.59, p<0.0001) and self-mentions (χ^2^ = 187.59, p<0.0001) to construct their stance in writing.

**Table 3:** Macro-categorical distribution of stance markers.

Attitude markers	Newspaper OC			Medical RA			χ2	P-value
	Raw frequency	Frequency per 1000 words	Percentage	Raw frequency	Frequency per 1000 words	Percentage		
Hedges	3833	20.3	36.0%	2530	13.5	40.5%	255.56	<0.0001
Boosters	3270	17.3	30.6%	1808	9.7	28.9%	408.76	<0.0001
Attitude markers	1200	6.3	11.2%	412	2.2	6.6%	376.59	<0.0001
Self-mentions	2373	12.5	22.2%	1501	8.0	24.0%	187.59	<0.0001
Total	10676	56.4	100%	6251	33.4	100.0%		

* The difference is significant at the 0.0001 level (P<0.0001).

### 3.2 Sub-categorical distribution of stance markers in newspaper OC and medical RA

As for category of hedges, modal verbs describing possibility (9.7 per 1000 words) and approximators (6.2 per 1000 words) in newspaper OC are more prevalent than those in medical RA, whereas epistemic lexical verbs (3.2 per 1000 words) and probability adverbs, adjectives and nouns (2.4 per 1000 words) in medical RA outnumber the frequencies of those items in newspaper OC (see Table 4). In terms of the subcategory like adverbs, adjectives and nouns expressing epistemic possibility, there is not any statistical difference between newspaper OC and medical RA (χ^2^ = 1.963, p = 0.16).

**Table 4:** Sub-categorical distribution and percentage of stance markers.

Stance markers		Newspaper OC			Medical RA			χ2	P-value
		Raw frequency	Frequency per 1000 words	Percentage	Raw frequency	Frequency per 1000 words	Percentage		
Hedges	Modal Verbs	1830	9.7	17.1%	954	5.1	15.3%	266.733	<0.0001
	Epistemic lexical verbs	420	2.2	4.0%	600	3.2	9.6%	33.660	<0.0001
	Adverbs, adjectives and nouns	413	2.2	3.9%	450	2.4	7.2%	1.963	0.16
	Approximators	1167	6.2	11.0%	526	2.8	8.4%	237.31	<0.0001
	Other phrases	3	0.0	0.0%	0	0.0	0.0%	1.310	0.25
Boosters	Modal verbs	806	4.3	7.5%	112	0.6	1.8%	516.254	<0.0001
	Epistemic lexical verbs	777	4.1	7.3%	685	3.7	10.9%	4.657	0.03
	Adverbs and adjectives	1521	8.0	14.2%	899	4.8	14.4%	153.08	<0.0001
	Attributors	123	0.6	1.2%	107	0.6	1.7%	0.810	0.37
	Other phrases	43	0.2	0.4%	5	0.0	0.0%	28.089	<0.0001
Attitude Markers	Deontic verbs	536	2.8	5.0%	136	0.7	2.2%	232.631	<0.0001
	Attitudinal adverbs	442	2.4	4.1%	85	0.5	1.4%	236.630	<0.0001
	Attitude adjectives	184	1.0	1.7%	191	1.0	3.1%	0.180	0.67
	Cognitive verbs	38	0.2	0.4%	0	0.0	0.0%	37.557	<0.0001
Self-mentions	First person pronouns	2373	12.5	22.2%	1476	7.9	23.6%	200.162	<0.0001
	Nouns phrases	0	0.0	0.0%	25	0.1	0.4%	25.299	<0.0001
Total		10676	56.4	100%	6251	33.4	100%		

* The difference is significant at the 0.0001 level (P<0.0001).

As shown in Table 4, there are far more boosters employed in newspaper OC than those used in medical RA. However, the differences are not statistically significant in terms of two subcategories: adverbs and adjectives expressing epistemic certainty (χ^2^ = 4.657, p = 0.03) and attributors (χ^2^ = 0.810, p = 0.37). One interesting finding in the category of boosters is that adverbs and adjectives expressing epistemic certainty dominate the distribution of boosters in both genres. Another more detailed examination of boosters reveals that the occurrences of modal verbs are far more frequent in newspaper OC than in medical RA (4.3 per 1000 words vs. 0.6 per 1000 words).

In terms of attitude markers, opinion columnists are apt to express their appraisal of propositions by deontic verbs (2.8 per 1000 words) and attitudinal adverbs (2.4 per 1000 words), whereas medical research writers would like to convey the importance of their research findings through attitude adjectives (1.0 per 1000 words) and deontic verbs (0.7 per 1000 words). Cognitive verbs are rarely used by medical researchers to convey their attitudes. Although attitudinal adjectives are employed by both columnists and researchers to construct their stance, there is not any statistical difference between these two genres (χ^2^ = 0.180, p = 0.67). Finally, large numbers of first person pronouns presenting self-mentions are found in both the sub-corpora. Given the special features of opinion articles, it is not surprising to find that there are not any noun phrases like *the writer* or *the author* used in newspaper OC.

A more detailed look into subcategories of stance markers displays, however, interesting similarities and differences from a cross-genre angle (see Table 4). The top three sub-categories of stance markers among the two genres are first person pronouns, modal verbs expressing possibility and adverbs and adjectives expressing certainty with the occurrences in newspaper OC outnumbering those in medical RA (12.5 per 1000 words vs. 7.9 per 1000 words, 9.7 per 1000 words vs. 5.1 per 1000 words, 8.0 per 1000 words vs. 4.8 per 1000 words). Furthermore, nouns and phrases in the category of self-mentions, cognitive verbs in attitude category and other phrases in booster category are relatively rare in both genres.

### 3.3 The ranked frequency of stance markers in newspaper OC and medical RA

In order to have a clear comparison of the distribution of stance markers between the two genres and find out the predominant stance markers in each category, a ranked frequency distribution of stance markers in both of the data seems very necessary. Table 5 demonstrates the top ten hedges in newspaper OC and medical RA. *Can* (3.00 per 1000 words) stands in the first place of hedges in newspaper OC, whereas *could* (1.19 per 1000 words) stands in the first place of hedges in medical RA. Among the top ten hedges in both the sub-corpora, modal verbs account for almost half of the top ten linguistic forms. Those tentative modal verbs are used to present claims and open a dialogic alternative with members of discourse community.

**Table 5:** The ranked frequency of the top ten hedges.

Newspaper OC			Medical RA		
Hedges	Raw frequency	Frequency per 1000 word	Hedges	Raw frequency	Frequency per 1000 word
Can	567	3.00	Could	222	1.19
About	506	2.67	Can	195	1.04
Would	388	2.05	Would	163	0.87
Could	338	1.79	Might	162	0.87
May	238	1.26	May	151	0.81
Might	148	0.78	Estimates (n.)	148	0.79
Around	131	0.69	Likely	145	0.78
Likely	116	0.61	Estimated (v.)	108	0.58
Can’t	100	0.52	About	97	0.52
Often	82	0.43	Possible	87	0.47

In addition to the modal verbs in Table 5, other frequently used linguistic forms include the sub-category of epistemic adjectives, nouns and approximators, such as *likely*, *possibly*, *estimates(n*.*)*, and *about*. In both sub-corpora, we see a more overt use of *about* (2.67 per 1000 words and 0.52 per 1000 words, respectively), which is typically used to express uncertainty on exact quantities or frequencies. The frequent use of word *about* may indicate the writer’s relative lack of knowledge over the issues discussed in the article. Rather than avoiding taking responsibilities of the statement or reducing the writer’s commitment towards the claim, quantifiers like *about* might suggest the writer’s genuine uncertainty about the truth value of the propositions conveyed in the article.

The results in Table 6 reveal the top ten boosters in both sub-corpora. In medical RA, most of the top ten boosters belong to the sub-category of epistemic lexical verbs, such as *showed*, *found*, *shown*, *known*, and *confirmed*. This result is consistent with discipline knowledge-making practice in medical research, in which most of the claims are made based on the objective observation of empirical data. However, the top ten boosters in newspaper OC demonstrate a great variety of linguistic features. Those linguistic devices for emphasizing certainty vary greatly from modal verbs to epistemic adverbs and adjectives, including modal verbs like *will* and *must*, adverbs like *never* and *especially*, adjectives like *clear*, *high*, and *great*.

**Table 6:** The ranked frequency of the top ten boosters.

Newspaper OC			Medical RA		
Boosters	Raw frequency	Frequency per 1000 word	Boosters	Raw frequency	Frequency per 1000 word
Will	692	3.66	High	253	1.35
Know	189	1.00	Showed	138	0.74
High	109	0.58	Found	132	0.71
Think	86	0.45	Will	109	0.58
Must	82	0.43	Total	104	0.56
Clear	82	0.43	Shown	99	0.53
Especially	81	0.43	According to	98	0.52
Never	71	0.38	Known	57	0.31
Great	71	0.38	Highly	53	0.28
Find	69	0.37	Confirmed	53	0.28

Attitude markers refer to the linguistic forms that writers comment on propositional contents or entities in the real world. They are essentially concerned with writers’ feelings, judgments, and affective positions. Table 7 shows the top ten attitude markers in both of the data. Given that the particular characteristics of newspaper OC is to express the columnist’s stance on an issue, it is not surprising to find that the frequency of attitude markers in OC is much more than those in medical RA. A close look at the top ten items in Table 7, however, shows that there are six overlapping attitude markers in both sub-corpora, including *even X*, *should*, *have to*, *important*, *appropriate*, and *unexpected*. These different sub-categories of attitude markers provide writers with an array of options to make evaluations of things in the real world.

**Table 7:** The ranked frequency of the top ten attitude markers.

Newspaper OC			Medical RA		
Attitude markers	Raw frequency	Frequency per 1000 word	Attitude markers	Raw frequency	Frequency per 1000 word
Even X	378	2.00	Should	130	0.70
Should	338	1.79	Important	95	0.51
Have to	118	0.62	Even X	61	0.33
Important	79	0.42	Expected	50	0.27
Must	73	0.39	Appropriate	14	0.08
Usual	23	0.12	Successfully	11	0.06
I think	23	0.12	Importantly	9	0.05
Appropriate	16	0.09	Striking	6	0.03
Unfortunately	15	0.08	Have to	5	0.03
Expected	14	0.07	Interestingly	4	0.02

Table 8 shows the most common self-mentions used in newspaper OC and medical RA. *I* stands in the first place in OC while *we* stands in the first place in RA. This can be easily explained by different genre features. Opinion articles are usually written by independent columnists, whereas the contributors to a single medical research often consist of many researchers, who play different roles in the whole research process. Thus, it is quite common to see a single author in an opinion article and multiple authors in a medical journal article. Besides the first person plural forms, medical research writers often use other linguistic forms to convey their presence in the research, like *authors*, *researchers*.

**Table 8:** The ranked frequency of the most common self-mentions.

Newspaper OC			Medical RA		
Self-mentions	Raw frequency	Frequency per 1000 word	Self-mentions	Raw frequency	Frequency per 1000 word
I	1572	8.31	We	1011	5.41
My	500	2.64	Our	447	2.39
Me	294	1.55	Us	18	0.10
Mine	7	0.03	The author	13	0.07
The author	0	0	Authors	10	0.05

## 4. Discussion

This study has revealed some key rhetorical differences in the construction of stance in newspaper OC and medical RA concerning the topic of COVID-19. The results also demonstrate that writers of different genres employ various linguistic resources of stance markers to achieve their rhetorical goals [12, 22, 36].

The results of this study show that stance markers are more frequently used in newspaper OC than medical RA. The results of chi-square tests regarding the overall use of stance markers confirm the statistical significance of the difference in the four categories of stance. The findings also demonstrate that opinion columnists tend to use more linguistic resources in persuasive writing. This is due to the fact that “the opinion section of U.S. newspapers usually refers to contexts where opinion is compartmentalized away from objective reporting” [54]. Journalistic reporters usually adhere to the objective norm of reporting and function as an arbiter of the truth and facts, whereas columnists mainly rely on their experiences or reputations to manifest their authority. They go beyond what happened in the world to the explanation of events in order to offer public readers a framework for evaluating events.

### 4.1 Hedges

The results regarding each category of stance markers reveal that hedges are the most frequently used category in both genres. Hedges in newspaper OC outnumber hedges in medical RA with modal verbs accounting for 17.1% and 15.3% of all stance markers in newspaper OC and research RA respectively. Those modal verbs represent the force of statements proposed by the writer and indicate the uncertainty when the information cannot be confirmed or verified. Due to the conventions of academic research, medical research writers are very cautious when they consider the amount of certainty projected into their statements, and thus modal verbs can function as shields for researchers to avoid absolute statements which might put them in a risky situation. Opinion columnists as insightful analysts and interpreters also need to use these rhetorical devices to project their discursive authority and build a relationship of trust between them and public readers, which is essential in producing successful persuasive writing.

In both sub-corpora, the most frequently used modal verbs are *could*, *can*, *would*, *might* and *may*, which account for 43.8% of hedges in OC and 29.4% of hedges in RA respectively. Opinion columnists use hedges to reduce the imposition of statements on readers in order to persuade them to believe in what is conveyed [22]. Those modal verbs are mainly concerned with the writer’s assessment or belief to status of the statement. The following examples 1–4 illustrate typical instances of such use.

(1) The upheaval in oil markets **could** also make a considerable dent in the American economy, Clifford Krauss, a national energy business correspondent for The Times, writes, especially as COVID-19 causes energy-intensive economic activity to slow. (The New York Times, March 10, 2020)

(2) Larger lenders **may** be less **likely** to cut jobs than small business borrowers, but they should also be compensated as the crisis abates. (The New York Times, March 24, 2020)

(3) Teachers and administrators **would** also have to be able to opt in, knowing they **could** acquire COVID-19. Colleges and universities **might** open up for summer sessions, with faculty and staff opting in, or not, with knowledge of the risks they are taking. (The New York Times, March 28, 2020)

(4) They must also address the concerns of those who fear that doctors **may** abuse their professional discretion under this protection of immunity. (The New York Times, April 4, 2020)

Myers [55] once stated that “researchers have to present themselves as the humble servants of the discipline, since claiming precision is not appropriate in all situations and writers do not always want to be precise.” Although medical writers’ scientific claims are based on the amounts of reliable data, they still seek to modify their assertions and avoid absolute claims which might put themselves in an embarrassing situation. Thus, researchers show a great deal of consideration to their statements, and keep their claims open to the alternative interpretations, as illustrated in examples 5–8.

A relative reduction of transmissibility **could** be achieved through early detection and isolation of cases, as well as behavioral changes and awareness of the disease in the population. (Science, March 6, 2020)

(6) Precisely what and how much should be done is highly contextually specific and there is no one-size-fits-all set of prescriptive interventions that **would** be appropriate across all settings. (The Lancet, January 31, 2020)

(7) Sepsis was a common complication, which **might** be directly caused by SARS-CoV-2 infection, but further research is needed to investigate the pathogenesis of sepsis in COVID-19 illness. (The Lancet, March 9, 2020)

(8) Therefore, older age related to death **may** be due to less robust immune responses. (JAMA, March 13, 2020)

Based on the above analysis of the modal verbs, we can see that modality could be regarded as a grammatical way to express writer’s attitudes and opinions [56]. However, the relatively indeterminate nature of modal meanings could not provide us a precise identification of their basic meanings. For example, modal verb *can* is often described in terms of possibility, ability and permission, which often brings more controversy than any other modal verbs [53]. Quirk et al. [57] argued that despite of the fact that *can* expresses ability which involves human control over event, “Ability is best considered a special case of possibility.” This treatment of *can* is highly inconsistent with our perception of *can* identified in the two sub-corpora. We believe that the meaning of *can* depends on its position in this possibility-ability continuum. As Palmer [58] suggests, “. . .there is no a priori reason why there should be a single meaning (of the modals); it is more likely that there is a conglomeration of vaguely related meanings. . .”. Since the line between these meanings could not be clearly cut, we regard the following concordance examples of *can* from both sub-corpora as expressing writer’s judgements about the state of affairs.

Availability of a public dataset enables independent estimation of important epidemiological parameters by several teams, allowing for confirmation and cross-checking at a time when information **can** be conflicting and noisy. (The Lancet, February 20, 2020)

Pharyngeal congestion is more like an upper respiratory symptom than is fever (which is a systemic symptom) and cough, which **can** also be induced by a lower respiratory infection. (The Lancet, March 25, 2020)

These estimates **can** be combined with estimates of the infection attack rate (approximately 80% for an unmitigated epidemic) to give rough projections of scale. (The Lancet, March 30, 2020)

The number of deaths seems like it should be easy enough to determine: After all, dead is dead. And yet ascribing a cause of death **can** be tricky. (The New York Times, February 18, 2020)

Interruptions in education **can** profoundly harm child development and make it harder to reduce the achievement gap between high- and low-income families. (The New York Times, March 10, 2020)

Small initial exposures tend to lead to mild or asymptomatic infections, while larger doses **can** be lethal. (The New York Times, April 1, 2020)

The modal verb *can* in example 9–11 allows medical researchers to convey their tentativeness and commitment, and leaves some room for other experts or discourse community members to make judgments and evaluations on the truth value of their statements. Across all medical texts in our corpus, modal verbs are the most frequently occurring stance markers compared with other types of hedges. The frequent use of modal verbs in medical RA further support previous findings that modal verbs are used to tone down the degree of certainty on author’s part as well as linked to the expression of precision or negative politeness [31, 32]. In addition, the modal verb *can* in newspaper examples 12–14 also deals with authors’ judgements about the state of affairs. This result is in contrast with Gray et al. [36] finding that *can* is used primarily to express ability. The explanation behind this high frequency of *can* expressing epistemic possibility in our corpora could be attributed to two aspects: first, the explanation framework adopted in our analysis regards the meaning of *can* as a continuum with possibility and ability at the opposite end. The interpretive meaning thus relies on readers’ perception of the events and contextual information to make sense of authors’ communicative purposes; second, the high frequency of *can* expressing the epistemic possibility is likely to be related with the topic of COVID-19. The research data in our corpora is all collected at the early stage of the pandemic outbreak and there is less known about the coronavirus disease. Not only medical researchers show their discretion when it comes to the explanation of research findings, but also newspaper opinion columnists are quite cautious in dealing with drawing definite conclusions about the topic. This further reinforces the communicative strategy of hedges to reduce the force of the statements by explicitly quantifying writers’ commitment [49].

Among the sub-categories of hedges, the chi-square test of epistemic adverbs, adjective and nouns reveals that there is not a significant difference between their usage in both genres. However, the linguistic forms of this sub-category differ greatly in both sub-corpora. As can be seen from Table 9, opinion columnists prefer to use modal adverbs while medical research writers tend to use epistemic nouns to modify statements. In newspaper OC, columnists use hedges to soft the statement and allow readers to make their own conclusions about the value of the proposition [22]. Those modal probability adverbs in example 15–17 such as *possibly*, *maybe*, *perhaps* appear in content disjunct function, which indicates the writer’s attitude towards the statement [32].

**Table 9:** The frequency of the most common epistemic adverbs, adjectives and nouns.

Newspaper OC			Medical RA		
Epistemic adverbs, adjectives and nouns	Raw frequency	Frequency per 1000 words	Epistemic adverbs, adjectives and nouns	Raw frequency	Frequency per 1000 words
Likely (adj.)	116	0.6	Estimates (n.)	148	0.8
Possible (adj.)	76	0.4	Likely (adj.)	145	0.8
Perhaps (adv.)	43	0.23	Possible (adj.)	87	0.47
Maybe (adv.)	40	0.21	Probability (n.)	65	0.35
Probably (adv.)	32	0.17	Estimate (n.)	57	0.31
Possibly (adv.)	23	0.12	Probably (adv.)	27	0.14
Estimates (n.)	14	0.07	Likelihood (n.)	25	0.13
Unlikely (adj.)	11	0.06	Assumption (n.)	22	0.12
Apparent (adj.)	9	0.05	Possibility (n.)	16	0.09
Essentially (adv.)	9	0.05	Probable (adj.)	14	0.08

(15) In the context of today’s viral threat, when millions of residents worry about who comes to the door and 1000s of census takers hesitate about putting themselves at risk, the census will weigh its options, **possibly** making greater use of administrative records than initially planned. (The New York Times, March 11, 2020)

(16) **Maybe** this gesture of solidarity will prove habit-forming—and worth continuing even when the virus recedes. (The New York Times, March 24, 2020)

(17) **Perhaps** the sensation of imprisonment during quarantine might make us imagine what real imprisonment feels like. (The New York Times, April 10, 2020)

In medical RA, the frequent use of epistemic nouns suggests the objective and impersonal features of academic writing. Medical research writers tend to make their claims sound more objective when stating speculative possibilities [36]. They use epistemic noun forms to distance themselves from the statements in order to shift readers’ attention to research findings rather than the person who presents the claim. Those epistemic nouns identified in examples 18–21 share a common feature of tentativeness towards the claims, which makes authors’ subjective views sound more objective.

These early **estimates** give an indication of the fatality ratio across the spectrum of COVID-19 disease and show a strong age gradient in risk of death. (The Lancet, March 30, 2020)

(19) At 80% of contacts traced, the **probability** of achieving control fell from 89% to 31%, with a long delay from onset to isolation. (The Lancet, February 28, 2020)

(20) We adapted an influenza epidemic simulation model to estimate the **likelihood** of human-to-human transmission of severe acute respiratory syndrome coronavirus2 (SARS-CoV-2) in a simulated Singaporean population. (The Lancet, March 23, 2020)

(21) Although we know of no reason to doubt specificity of detection, we tested the **assumption** of 100% sensitivity. (The Lancet, April 1, 2020)

### 4.2 Boosters

Compared with frequency of hedges in both genres, boosters are used less frequently but are still a necessary means of achieving rhetorical goals. Opinion columnists use more boosters than medical research writers to enhance the force of their statements. The results of boosters show that there are about 17.28 boosters per 1000 words in newspaper OC while there are 9.9 boosters per 1000 words in medical RA. The frequency of modal verbs and the frequency of adverbs and adjectives of certainty reveal statistically significant differences between two sub-corpora, but writers of different genres employ different linguistic options to realize their rhetorical goals.

The top ten boosters in newspaper OC (see Table 6) include only three epistemic verbs *know*, *think* and *find*, which together account for 44% of all epistemic lexical verbs of booster category used in newspaper genre. Besides these lexical verbs, opinion columnists have various linguistic forms of boosters to draw on, such as adjectives *clear*, *great* and adverb *never* which are among the most frequent boosters in newspaper OC. This is probably related with the communicative purpose of opinion discourse, which indicates opinion columnists try to persuade readers with the great amount of certainty put in the proposition and leave little room for a reader’s objection. The following examples 22–24 illustrate the features of boosters used in newspaper OC.

We **know** from flu research that mask-wearing can help decrease transmission rates along with frequent hand-washing and social-distancing. (The New York Times, March 17, 2020)

(23) At the same time, other people have **never** been more dangerous to our health. The choice to go outside, even to help others, means assuming risk—for yourself, loved ones and strangers. (The New York Times, March 23, 2020)

(24) That is **clear** from a recent Times article that quoted a Silicon Valley start-up adviser who called this “the great unwinding”—a perfectly apt term. (The New York Times, April 8, 2020)

Myers [59] regard boosting as positive-politeness devices, which allow writers to negotiate the status of their statements and stress common group membership by strategic expressions. The main purpose of medical researchers is to persuade the discourse community members to accept what they have proposed, and it is crucial for researchers to make an appropriate level of their claims [60]. According to Ngai et al. [61], scientists from life science employ more boosters for persuasion and the lexical verbs *show*, *find*, *demonstrat*e are proven to be frequently used boosters in article abstracts, which is highly consistent with our findings that lexical verbs such as *showed*, *found*, *shown*, *known* and *confirmed* account for almost 70% of all epistemic lexical verbs of booster category used in medical RA. The following examples 25–27 illustrate typical instances of boosters in medical RA.

Follow-up images showed obviously resolved bilateral ground glass opacity and consolidation. (BMJ, March 26, 2020)

(26) This study found that the implementation of public health interventions was associated with a reduction in Rt to below 1.0 on February 6 and to below 0.3 on March 1, which may have implications for global efforts to contain the pandemic more broadly. (JAMA, April 10, 2020)

(27) Studies demonstrated that T-cell responses can inhibit the over- activation of innate immunity. (JAMA, March 13, 2020)

In our study, attributors are also classified as a sub-category of boosters and the linguistic forms used in the realization of attribution of information do not differ greatly from each genre. The chi-square test of attributors shows no significant difference (χ^2^ = 0.810, p = 0.37) between the two genres. The rhetorical strategy of attribution in newspaper OC is to confirm the truth of information by virtue of credibility of the source of information [62], while medical RA writers often employ attributors to establish the intertextuality through the reference of other writers. Both writers of the two genres rely on phrase *according to* and conjunction *as* to provide evidence to support their arguments or establish the intertextuality. Those linguistic resources can guide readers to assess the truth value of the proposition as writers would wish, which is illustrated in examples 28–31.

(28) **According to the American Thoracic Society guidelines** for community-acquired pneumonia, 1088 patients (41.1%) had severe infection and 126 patients (58.9%) had nonsevere infection. (JAMA, April 10, 2020)

(29) **As previously reported**, patients with a history of cerebrovascular disease are at increased risk of becoming critically ill or dying if they have SARS-CoV-2 infection. (The Lancet, February 21, 2020)

(30) But in highly unequal societies, such as ours, wealth is no protection from illness, **according to a series of studies conducted by two British epidemiologists**, Richard G. Wilkinson and Kate Pickett. (The New York Times, March 20, 2020)

(31) That’s the technical term for tracking down anybody who has come in contact with a person who’s newly diagnosed with the virus. It is, **as Caitlin Rivers of Johns Hopkins University says,** “very laborious.” (The New York Times, April 10, 2020)

### 4.3 Attitude markers

Compared with hedges and boosters, attitude markers are the least frequent stance markers used in both sub-corpora. Regarding that the major purpose of newspaper OC is to express the columnist’s attitude on a topic, it is not unexpected to find that the normal frequency of attitude markers in newspaper OC (6.3 per 1000 words) are nearly three times as frequent as those in medical RA (2.2 per 1000 words). The explicit attitude markers allow authorial intrusion into the texts, which is against the knowledge construction practice of natural science [22].

Writers use attitude markers not only to express their stance towards a proposition, but also to share with readers the feelings and assessments they are presenting, thus declaring writers’ attitudes in a dialogical way with the purpose of establishing the alignment with addressees about the shared belief and value [63]. Newspaper OC readers may hold different views when they engage themselves with the column. This might explain why opinion columnists are more likely to employ a greater range and diversity of attitude adverbials to engage their readers. However, compared with [22], the range of attitude adverbs and adjectives employed in our newspaper OC corpus is relatively small and limited, and this could be explained by our special focus on the topic of COVID-19. The following examples 32–37 illustrate the typical instances of attitude markers in both genres.

(32) In a normal recession, monetary and fiscal stimulus might be **appropriate**. (The New York Times, March 24, 2020)

(33) And if a certain line of argument is bad—as **I think**, and argued last week, that the right-wing anti-lockdown argument is bad—then it has to be judged on its own merits, not just dismissed because it lacks the C.D.C.’s patina. (The New York Times, April 7, 2020)

(34) **Unfortunately**, the $2 trillion COVID-19 stimulus package passed last month included only $11 billion that H.H.S. can use for patent buyouts, and the department will most likely need to draw down some of those funds for other purposes, like procuring diagnostic tests and purchasing other medical equipment. (The New York Times, April 8, 2020)

(35) Molecular techniques have been used **successfully** to identify infectious agents for many years. (NEJM, March 26, 2020)

(36) **Importantly**, this virus sequence, obtained from a pangolin scale sample, may in fact be derived from contaminants of other infected tissues. (Nature, March 26, 2020)

(37) **Successful** and **appropriate** use of the App relies on it commanding well-founded public trust and confidence. (Science, March 31, 2020)

As for deontic verbs in our study, the normal frequency of deontic verbs in newspaper OC (2.8 per 1000 words) are four times as frequent as those in medical RA (0.7 per 1000 words). Khabbazi-Oskouei [64] made a distinction between the various targets of the expressions of obligation. He emphasized that when the addressee of the obligation markers such as *should*, *must*, *have to* is the third part (not including the reader), those deontic verbs can be regarded as attitude markers. These deontic verbs demonstrate how the writer intervenes in the discourse to highlight the necessity of actions to be taken. According to Vihla [65], deontic expressions are related with “reference to a moral, legal or professional code” and imply “the existence of an authority having the power to say what is right or wrong”. In our study, the deontic expressions like *should*, *have to* and *must* are among the top ten attitude markers in newspaper OC, whereas only deontic verb *should* is ranked the most frequent attitude marker in medical RA (see Table 7) and there is only a few examples concerning the usage of *must* in our medical corpus. The deontic verbs in the following examples 38–42 are used to point out an obligation or propose a suggestion for the action to be taken in the given circumstance.

(38) If schools close, child care programs will likely close too and working parents may **have to** stay home to watch their children. (The New York Times, March 10, 2020)

(39) Courts **should** also explore increasing access for people to pay fines for traffic offenses or violations of municipal ordinances by mail or online. (The New York Times, March 13, 2020)

(40) Free-market true believers—including officials in the Trump administration—argue that pharmaceutical businesses **must** be allowed to set prices beyond some patients’ reach. (The New York Times, April 8, 2020)

(41) Therefore these findings **should** be combined with the latest risk assessments that are available for COVID-19 and other assessments to understand the existing capacities. (The Lancet, March 18, 2020)

(42) Using the current best understanding, around 80% of symptomatic contacts **must** be traced and isolated to control over 80% of outbreaks in the model. (The Lancet, February 28, 2020)

### 4.4 Self-mentions

Despite the fact that the chi-square test of self-mentions shows some statistically significant differences between newspaper OC and medical RA, the linguistic forms used to realize the writer’s presence in both genres vary greatly from each other. The findings reveal that the first person singular forms such as *I*, *me*, *my* and *mine* in newspaper OC account for the whole frequency of self-mentions (12.5 per 1000 words), whereas in medical RA the first person plural forms such as *we*, *us* and *our* are the dominant linguistic forms (7.9 per 1000 words). In addition to those first person pronouns, a small number of noun phrases are also adopted by medical researchers to express their identity. The employment of self-mentions in both genres indicates that writers have a great tendency to attach themselves to the discourse community and present propositional, affective and interpersonal information [66].

In our findings, we have found a large number of first person plural forms highlighting writers’ presence in medical RA. Ivanič [67] pointed out that presenting a discourse self is central to the writing process. As examples 43–44 illustrate, such explicit personal projection into the medical texts seeks to emphasize their contribution into the research field and reach the agreement with discourse community members. In newspaper OC, however, the writer’s stance is more personal, and there is a great need for them to establish an intimate relationship with readers and promote the credibility of arguments. Therefore, self-mentions in examples 45–46 carry a greater weight to bring the author’s voice to an issue and seek the agreement between readers’ views and authors’ own.

(43) **We** aimed to provide robust estimates, accounting for censoring and ascertainment biases. (Lancet, March 30, 2020)

(44) Overall, the novelty of **our** approach was to rely on a unique source for social media and news reports in China, which aggregated and curated relevant information. (Lancet, February 20, 2020)

(45) That by nighttime, **my** anger had melted away to tears, leaving **me** collapsed on the couch. (The New York Times, March 7, 2020)

(46) One challenge **I**’m noticing is how to manage the influx of out-of-state visitors and residents coming into the state. (The New York Times, April 10, 2020)

## 5. Conclusion

The present study sets out to compare the frequency of categories of stance markers in newspaper OC and medical RA concerning the same topic with the aim of finding out similarities and differences of the distribution of stance markers in the two genres. The most frequent category of stance markers is hedges in both newspaper OC and medical RA, demonstrating the writer’s caution and modesty in making claims and also the necessity of expressing proper precision [7]. The least frequent category of stance markers in each genre is attitude markers. The reason behind the less usage could be attributable to the fact that the contents of the corpus focus on COVID-19. Writers of both genres are very cautious and they may be reluctant to make their opinions explicit when dealing with the topic of COVID-19 pandemic, and this reluctance might be explained by the fact that there is not much known to the coronavirus during the early period of the outbreak. Although the frequency of stance markers in newspaper OC is significantly greater than those in medical RA, there is no significant difference among the frequency of some sub-categories. For example, further analysis of the sub-categories such as epistemic adverbs, adjectives and nouns of hedges, epistemic lexical verbs and attributors expressing certainty, and attitude adjectives reveals less distinct pattern of results.

In addition, the study also demonstrates that although there exists significant differences of the frequency of stance markers in newspaper OC and medical RA, the distribution of those markers in each genre indicates some overlapping among their usage, especially among the top ten stance markers in each category. This further reflects the fact that the construction of stance is highly related to the topic and conventions in different discursive practice. Genre is considered as a social construct with certain communicative purpose, and writers resort to different linguistic resources to realize their rhetorical goals. However, the topic content seems to play an important role in shaping the way of how writers construct their stance. The lack of adequate scientific information or evidence on COVID-19 during the early period of the pandemic could restrain writers from making high degree of commitment to their claims, which makes them adopt a more tentative stance by using more hedging expressions to qualify their statements.

Based on what we have found so far, there are also some limitations in the present research. The newspaper data was collected only from one mainstream newspaper and more diversified newspapers should be considered during the compilation of corpus in order to make the data more representative. The analysis of the stance markers was based on pre-selected linguistic features, which may render interpretation of results subjective. We recommend that future research may use a corpus-driven approach to show what to investigate further, and uncover new stance constructions through inductive analysis of corpora. Another limitation for this study is the small size of the two sub-corpora, which makes it impossible to gather enough valuable evidence for the stance collocates of COVID-19. In addition, this study uses different sub-corpora as observations to compute rates of occurrence of linguistics features. Future study may adopt a different research design and treat each text as the unit of analysis, thus providing numeric variables for inferential statistical tests. By considering different approaches, researchers could identify some linguistics features which are interesting for exploration.

## Supporting information

S1 File (ZIP)
