# Supplementary material for: Stance markers in English medical research articles and newspaper opinion columns: A comparative corpus-based study
Source: PLoS One. 2021 Mar 8;16(3):e0247981. doi: 10.1371/journal.pone.0247981 (PMC7939291; doi:10.1371/journal.pone.0247981)
Supplement: S1 File — (ZIP) [file pone.0247981.s001.zip › Supporting information/supporting information 1/Supporting information 1-List of 52 Medical research articles.docx]

**The list of 52 medical research articles in seven medical journals**

**The names of seven medical journals:**

*The* *Lancet (Lancet), Science, Nature, The British Medical Journal (BMJ), The New England Journal of Medicine (NEJM), Proceedings of the National Academy of Sciences of the United States of America (Pans)* and *The journal of the America Medical Association (JAMA)*.

**Titles of research articles chosen from these seven journals:**

**1-BMJ**

Clinical findings in a group of patients infected with the 2019 novel coronavirus (SARS-Cov-2) outside of Wuhan,China:retrospective case series

PMID: 32075786  <https://doi.org/10.1136/bmj.m606>

**2-BMJ**

Clinical characteristics of 113 deceased patients with coronavirus disease 2019 retrospective study

PMID: 32217556 <https://doi.org/10.1136/bmj.m1091>

**3-Lancet**

Nowcasting and forecasting the potential domestic and international spread of the 2019-nCoV outbreak originating: a modelling study

PMID: 32014114 [https://doi.org/10.1016/S0140-6736(20)30260-9](https://doi.org/10.1016/S0140-6736(20)30260-9)

**4-Lancet**

A familial cluster of pneumonia associated with the 2019 novel coronavirus indicating person-to-person transmission：a study of a family cluster

PMID: 31986261 [https://doi.org/10.1016/S0140-6736(20)30154-9](https://doi.org/10.1016/S0140-6736(20)30154-9)

**5-Lancet**

Clinical features of patients infected with 2019 novel coronavirus in Wuhan, China

PMID: 31986264 [https://doi.org/10.1016/S0140-6736(20)30183-5](https://doi.org/10.1016/S0140-6736(20)30183-5)

**6-Lancet**

Epidemiological and clinical characteristics of 99 cases of 2019 novel coronavirus pneumonia in Wuhan, China: a descriptive study

PMID: 32007143 [https://doi.org/10.1016/S0140-6736(20)30211-7](https://doi.org/10.1016/S0140-6736(20)30211-7)

**7-Lancet**

Early epidemiological analysis of the coronavirus disease 2019 outbreak based on crowdsourced data

PMID: 32309796 [https://doi.org/10.1016/S2589-7500(20)30026-1](https://doi.org/10.1016/S2589-7500(20)30026-1)

**8-Lancet**

Clinical course and outcomes of critically ill patients with SARS-CoV-2 pneumonian Wuhan, China: a single-centered, retrospective, observational study

PMID: 32105632 [https://doi.org/10.1016/S2589-7500(20)30026-1](https://doi.org/10.1016/S2589-7500(20)30026-1)

**9-Lancet**

Genomic characterisation and epidemiology of 2019 novel coronavirus : implications for virus origins and receptor binding

PMID: 32007145 [https://doi.org/10.1016/S0140-6736(20)30251-8](https://doi.org/10.1016/S0140-6736(20)30251-8)

**10-Lancet**

Clinical characteristics and intrauterine vertical transmission potential of COVID-19 infection in nine pregnant women: a retrospective review of medical records

PMID: 32151335 [https://doi.org/10.1016/S0140-6736(20)30360-3](https://doi.org/10.1016/S0140-6736(20)30360-3)

**11-Lancet**

Early dynamics of transmission and control of COVID-19: a mathematical modelling study

PMID: 32171059 [https://doi.org/10.1016/S1473-3099(20)30144-4](https://doi.org/10.1016/S1473-3099(20)30144-4)

**12-Lancet**

First known person-to-person transmission of severe acute respiratory syndrome coronavirus 2 in the USA

PMID: 32178768 [https://doi.org/10.1016/S0140-6736(20)30607-3](https://doi.org/10.1016/S0140-6736(20)30607-3)

**13-Lancet**

Preparedness and vulnerability of African countries against importations of COVID-19: a modelling study

PMID: 32087820 [https://doi.org/10.1016/S0140-6736(20)30411-6](https://doi.org/10.1016/S0140-6736(20)30411-6)

**14-Lancet**

Interventions to mitigate early spread of SARS-CoV-2 in Singapore: a modelling study

PMID: 32213332 [https://doi.org/10.1016/S1473-3099(20)30162-6](https://doi.org/10.1016/S1473-3099(20)30162-6)

**15-Lancet**

Temporal profiles of viral load in posterior oropharyngeal saliva samples and serum antibody responses during infection by SARS-CoV-2: an observational cohort study

PMID: 32213337 [https://doi.org/10.1016/S1473-3099(20)30196-1](https://doi.org/10.1016/S1473-3099(20)30196-1)

**16-Lancet**

Clinical features and obstetric and neonatal outcomes of pregnant patients with COVID-19 in Wuhan, China: a retrospective, single-centre, descriptive study

PMID: 32220284 [https://doi.org/10.1016/S1473-3099(20)30176-6](https://doi.org/10.1016/S1473-3099(20)30176-6)

**17-Lancet**

Clinical and epidemiological features of 36 children with coronavirus disease 2019 (COVID-19) in Zhejiang, China: an observational cohort study

PMID: 32220650 [https://doi.org/10.1016/S1473-3099(20)30198-5](https://doi.org/10.1016/S1473-3099(20)30198-5)

**18-Lancet**

The effect of control strategies to reduce social mixing on outcomes of the COVID-19 epidemic in Wuhan, China: a modelling study

PMID: 32220655 [https://doi.org/10.1016/S2468-2667(20)30073-6](https://doi.org/10.1016/S2468-2667(20)30073-6)

**19-Lancet**

Clinical course and risk factors for mortality of adult inpatients with COVID-19 in Wuhan, China: a retrospective cohort study

PMID: 32171076 [https://doi.org/10.1016/S0140-6736(20)30566-3](https://doi.org/10.1016/S0140-6736(20)30566-3)

**20-Lancet**

Health security capacities in the context of COVID-19 outbreak: an analysis of International Health Regulations annual report data from 182 countries

PMID: 32199075 [https://doi.org/10.1016/S0140-6736(20)30553-5](https://doi.org/10.1016/S0140-6736(20)30553-5)

**21-Lancet**

Investigation of three clusters of COVID-19 in Singapore: implications for surveillance and response measures

PMID: 32192580 [https://doi.org/10.1016/S0140-6736(20)30528-6](https://doi.org/10.1016/S0140-6736(20)30528-6)

**22-Lancet**

Estimates of the severity of coronavirus disease 2019 : a model-based analysis

PMID: 32240634 [https://doi.org/10.1016/S1473-3099(20)30243-7](https://doi.org/10.1016/S1473-3099(20)30243-7)

**23-Lancet**

Feasibility of controlling COVID-19 outbreaks by isolation of cases and contacts

PMID: 32119825 [https://doi.org/10.1016/S2214-109X(20)30074-7](https://doi.org/10.1016/S2214-109X(20)30074-7)

**24-Lancet**

Radiological findings from 81 patients with COVID-19 pneumonia in Wuhan, China: a descriptive study

PMID: 32105637 [https://doi.org/10.1016/S1473-3099(20)30086-4](https://doi.org/10.1016/S1473-3099(20)30086-4)

**25-Lancet**

Using observational data to quantify bias of traveller-derived COVID-19 prevalence estimates in Wuhan, China

PMID: 32246905 [https://doi.org/10.1016/S1473-3099(20)30229-2](https://doi.org/10.1016/S1473-3099(20)30229-2)

**26-Nature**

A new coronavirus associated with human respiratory disease in China

PMID: 32015508 <https://doi.org/10.1038/s41586-020-2008-3>

**27-Nature**

A pneumonia outbreak associated with a new coronavirus of probable bat origin

PMID: 32015507 <https://doi.org/10.1038/s41586-020-2012-7>

**28-Nature**

Identifying SARS-CoV-2 related coronaviruses in Malayan pangolins

PMID: 32218527 <https://doi.org/10.1038/s41586-020-2169-0>

**29-Nature**

Structural basis of receptor recognition by SARS-CoV-2

PMID: 32225175 <https://doi.org/10.1038/s41586-020-2179-y>

**30-Nature**

Structure of the SARS-CoV-2 spike receptor-binding domain bound to the ACE2 receptor

PMID: 32225176 <https://doi.org/10.1038/s41586-020-2180-5>

**31-Nature**

Virological assessment of hospitalized patients with COVID-2019

PMID: 32235945 <https://doi.org/10.1038/s41586-020-2196-x>

**32-NEJM**

A Novel Coronavirus from Patients with Pneumonia in China, 2019

PMID: 31978945 <https://doi.org/10.1056/NEJMoa2001017>

**33-NEJM**

First Case of 2019 Novel Coronavirus in the United States

PMID: 32004427 <https://doi.org/10.1056/NEJMoa2001191>

**34-NEJM**

Early Transmission Dynamics in Wuhan, China, of Novel Coronavirus–Infected Pneumonia

PMID: 31995857 <https://doi.org/10.1056/NEJMoa2001316>

**35-PNAS**

Impact of international travel and border control measures on the global spread of the novel 2019 coronavirus outbreak

PMID: 32170017 <https://doi.org/10.1073/pnas.2002616117>

**36-PNAS**

Enhanced isolation of SARS-CoV-2 by TMPRSS2-expressing cells

PMID: 32165541 <https://doi.org/10.1073/pnas.2002589117>

**37-PNAS**

Projecting hospital utilization during the COVID-19 outbreaks in the United States

PMID: 32245814 <https://doi.org/10.1073/pnas.2004064117>

**38-Science**

The effect of human mobility and control measures on the COVID-19 in China

PMID: 32511452 <https://doi.org/10.1126/science.abb4218>

**39-Science**

Structural basis for the recognition of the SARS-CoV-2 by full-length human ACE2

PMID: 32132184 <https://doi.org/10.1126/science.abb2762>

**40-Science**

The effect of travel restrictions on the spread of the 2019 novel coronavirus (COVID-19) outbreak

PMID: 32144116 <https://doi.org/10.1126/science.aba9757>

**41-Science**

Substantial undocumented infection facilitates the rapid dissemination of novel coronavirus (SARS-CoV2)

PMID: 32179701 <https://doi.org/10.1126/science.abb3221>

**42-Science**

Quantifying SARS-CoV-2 transmission suggests epidemic control with digital contact tracing

PMID: 32234805 <https://doi.org/10.1126/science.abb6936>

**43-JAMA**

Epidemiologic Features and Clinical Course of Patients Infected With SARS-CoV-2 in Singapore

PMID: 32125362 <https://doi.org/10.1001/jama.2020.3204>

**44-JAMA**

Association of Cardiac Injury With Mortality in Hospitalized Patients With COVID-19 in Wuhan, China

PMID: 32211816 <https://doi.org/10.1001/jamacardio.2020.0950>

**45-JAMA**

Neurologic Manifestations of Hospitalized Patients With Coronavirus Disease 2019 in Wuhan, China

PMID: 32275288 [https://doi.org/10.1001/jamaneurol.2020.1127](https://doi.org/10.1001/jamaneurol.2020.1127 )

**46-JAMA**

Factors Associated With Mental Health Outcomes Among Health Care Workers Exposed to Coronavirus Disease 2019

PMID: 32202646 <https://doi.org/10.1001/jamanetworkopen.2020.3976>

**47-JAMA**

Risk Factors Associated With Acute Respiratory Distress Syndrome and Death in Patients With Coronavirus Disease 2019 Pneumonia in Wuhan, China

PMID: 32167524 <https://doi.org/10.1001/jamainternmed.2020.0994>

**48-JAMA**

Cardiovascular Implications of Fatal Outcomes of Patients With Coronavirus Disease 2019 (COVID-19)

PMID: 32219356 <https://doi.org/10.1001/jamacardio.2020.1017>

**49-JAMA**

Presenting Characteristics, Comorbidities, and Outcomes Among 5700 Patients Hospitalized With COVID-19 in the New York City Area

PMID: 32320003 [https://doi.org/10.1001/jama.2020.6775](https://doi.org/10.1001/jama.2020.6775 )

**50-JAMA**

Association of Public Health Interventions With the Epidemiology of the COVID-19 Outbreak in Wuhan, China

PMID: 32275295 [https://doi.org/10.1001/jama.2020.6130]( https:/doi.org/10.1001/jama.2020.6130)

**51-JAMA**

Baseline Characteristics and Outcomes of 1591 Patients Infected With SARS-CoV-2 Admitted to ICUs of the Lombardy Region, Italy

PMID: 32250385 <https://doi.org/10.1001/jama.2020.5394>

**52-JAMA**

Effect of High vs Low Doses of Chloroquine Diphosphate as Adjunctive Therapy for Patients Hospitalized With Severe Acute Respiratory Syndrome Coronavirus 2 (SARS-CoV-2) Infection A Randomized Clinical Trial

PMID: 32330277 <https://doi.org/10.1001/jamanetworkopen.2020.8857>
