# Supplementary material for: Stance markers in English medical research articles and newspaper opinion columns: A comparative corpus-based study
Source: PLoS One. 2021 Mar 8;16(3):e0247981. doi: 10.1371/journal.pone.0247981 (PMC7939291; doi:10.1371/journal.pone.0247981)
Supplement: S1 File — (ZIP) [file pone.0247981.s001.zip › Supporting information/supporting information 2/Supporting information 2-List of 175 opinion columns in The New York Times.docx]

Titles of 175 opinion columns articles from *The New York Times*

1. China’s Coronavirus Crisis Is Just Beginning
2. Come Back, New York, All Is Forgiven
3. Congress Needs a Plan to Confront the Coronavirus. I Have One
4. Coronavirus and the Isolation Paradox
5. Coronavirus School Closings: Don’t Wait Until It’s Too Late
6. Coronavirus Shows That We Have a Solidarity Problem
7. Coronavirus Will Test Our New Way of Life
8. Coronavirus： Revenge of the Pangolins?
9. Covid-19 and the Big Government Problem
10. Covid-19 Brings Out All the Usual Zombies
11. Covid-19 Is Twisting 2020 Beyond All Recognition
12. Covid-19： A Look Back From 2025
13. Doctors Need Room to Make the Wrenching Decisions They Face
14. Dogs, at Least, Love Home Quarantine
15. Don’t Blame New Orleans, and Don’t Forget It
16. Drug Companies Will Make a Killing From Coronavirus
17. Economic Stimulus Is the Wrong Prescription
18. Feeling Powerless About Coronavirus？ Join a Mutual-Aid Network
19. Finding the ‘Common Good’ in a Pandemic
20. Five Signs of Failure
21. For 12 Days a Doctor Lived With Burning Lungs
22. Fourteen Days. That’s the Most Time We Have to Defeat Coronavirus.
23. Fox’s Fake News Contagion
24. God Doesn’t Want Us to Sacrifice the Old
25. Google Searches Can Help Us Find Emerging Covid-19 Outbreaks
26. Halfhearted Millennial Coronavirus Prep in a New York City Apartment
27. He Got Tested for Coronavirus. Then Came the Flood of Medical Bills.
28. Heartache in the Hot Zone： The Front Line Against Covid-19
29. How Are We Supposed to Vote During a Pandemic？
30. How Bad Will the Coronavirus Outbreak Get？
31. How Can You Social Distance When You Share a Toilet With Your Neighbor？
32. How the Coronavirus May Force Doctors to Decide Who Can Live and Who Dies
33. How to Be a Smart Coronavirus Prepper
34. How to Confront the Coronavirus at Every Level
35. How to Fix the Coronavirus Testing Mess in 7 Days
36. How to Get Money to Small Businesses, Fast
37. How to Save Black and Hispanic Lives in a Pandemic
38. I Followed the U.K.’s Advice. Did I Spread Coronavirus？
39. I Hope My Neighbor Plays Beyoncé Tonight
40. I Live in Nebraska. We Need to Shelter-In-Place.
41. I Refuse to Run a Coronavirus Home School
42. I’m 26. Coronavirus Sent Me to the Hospital.
43. I’m a Doctor at the ‘Epicenter of the Epicenter’
44. I’m a Doctor in Britain. We’re Heading Into the Abyss.
45. I’m a Doctor in Italy. We Have Never Seen Anything Like This.
46. I’m Chinese. That Doesn’t Mean I Have Coronavirus.
47. I’m on the Front Lines. I Have No Plan for This.
48. I’ve Been in Prison. Sheltering in Place Is Terrifying.
49. In the American South, a Perfect Storm Is Gathering
50. In the Fog of Coronavirus, There Are No Experts
51. In This Emergency, Mom Knows Best
52. Is It a Pandemic Yet？
53. Is Our Fight Against Coronavirus Worse Than the Disease？
54. Is This Really the Best Way to Fight Coronavirus？
55. Is Your Grocery Delivery Worth a Worker’s Life？
56. It Took Me 3 E.R. Visits to Get a Coronavirus Test in New York
57. It’s Going to Be Difficult
58. It’s Time for the Business Community to Step Up
59. It’s Time to Declare a National Emergency
60. It’s Time to Talk About Death
61. It’s Too Late to Avoid Disaster, but There Are Still Things We Can Do
62. Japan Can’t Handle the Coronavirus. Can It Host the Olympics？
63. Jobs Aren’t Being Destroyed This Fast Elsewhere. Why Is That？
64. Just Use ‘the Computer’ at the Fed to Give People More Money
65. Lessons From Lockdown
66. Life in Seattle, America’s Coronavirus Capital
67. Listen. Relax. Sleep Can Boost Your Immunity.
68. Lockdown Can’t Last Forever. Here’s How to Lift It.
69. Markets Are Spooked. How Bad Could It Get？
70. My Father Didn’t Have Covid-19, but He Almost Died Because of It
71. My Life Is More ‘Disposable’ During This Pandemic
72. New York’s Paramedics, on the Front Lines and Forgotten
73. No Masks, Disinfectant or Soap. This Is Detention Amid a Pandemic.
74. Not Winning This Fight
75. Nothing Matters Anymore (Except What Actually Does)
76. On Coronavirus Lockdown？ Look for Meaning, Not Happiness
77. On Recklessness and the Coronavirus
78. Our Courts and Jails Are Putting Lives at Risk
79. Our New Historical Divide： B.C. and A.C. — the World Before Corona and the World After
80. Paranoid Politics Goes Viral
81. Pay Your House Cleaner Anyway
82. Pharmaceutical Profits and Public Health Are Not Incompatible
83. Phone Sex Is Safe Sex
84. Please, Don’t Go Out to Brunch Today
85. Please, Don’t Intentionally Infect Yourself. Signed, an Epidemiologist.
86. Prisons Breed the Coronavirus. We Can Safely Free Thousands of Inmates.
87. Protect the Doctors and Nurses Who Are Protecting Us
88. Quarantine the Sick in New York’s Hotels
89. Republicans Want Medicare for All, but Just for This One Disease
90. Rules for Using the Sidewalk During the Coronavirus
91. Seattle Is Living Your Coronavirus Future
92. Seven Important Things to Know About Coronaviruses
93. She’s Been a Nurse for 10 Years. Panic Has Finally Arrived.
94. Social Distancing Is a Privilege
95. Surviving Coronavirus as a Broke College Student
96. Testing is Just the Beginning in the Battle Against Covid-19
97. The America We Need
98. The Beautiful World Beside the Broken One
99. The Christian Response to the Coronavirus： Stay Home
100. The Coronavirus and the Conservative Mind
101. The Coronavirus Becomes an Excuse to Restrict Abortions
102. The Coronavirus Could Cause a Child Abuse Epidemic
103. The Coronavirus Could Change the Way We Take the Census
104. The Coronavirus Is Showing Us Which Entrepreneurs Matter
105. The Covid-19 Slump Has Arrived
106. The Epic Failure of Coronavirus Testing in America
107. The Ideas That Won’t Survive the Coronavirus
108. The Leaders Who Passed the Coronavirus Test
109. The Magic of Empty Streets
110. The Men and Women Who Run Toward the Dying
111. The Military Should Airlift New York City’s Coronavirus Patients
112. The Pandemic of Fear and Agony
113. The Pandemic’s Missing Data
114. The Racial Time Bomb in the Covid-19 Crisis
115. The Real Tragedy of Not Having Enough Covid-19 Tests
116. The Single Most Important Lesson From the 1918 Influenza
117. The Surprising Intimacy of the Live-Streamed Funeral
118. The U.S. Approach to Public Health： Neglect, Panic, Repeat
119. The Unholy Alliance of Trump and Dr. Oz
120. The United States Needs a ‘Smart Quarantine’ to Stop the Virus Spread Within Families
121. The Virus Comes for Democracy
122. The World Is Empty Now. How Should We Fill It？
123. There Is No Way Out but Through
124. There’s a Giant Hole in Pelosi’s Coronavirus Bill
125. These Coronavirus Exposures Might Be the Most Dangerous
126. They Don’t Hide From the Coronavirus, They Confront It
127. They’ve Contained the Coronavirus. Here’s How.
128. This Is the Stimulus We Need Right Now. It’s Not $1,000 for Every American.
129. This Won’t End for Anyone Until It Ends for Everyone
130. To Protect Global Health, Work With China
131. Trump Doesn’t Have the Attention Span to Fight Coronavirus
132. Trump Is Gutting Our Democracy While We’re Dealing With Coronavirus
133. Trump Keeps Putting the Lives of Lupus Patients at Risk
134. Trump Wants to ‘Reopen America.’ Here’s What Happens if We Do.
135. Virus as Metaphor
136. Voting by Mail Will Save the 2020 Election
137. We Can Safely Restart the Economy in June. Here’s How.
138. We Don’t Need to Close Schools to Fight the Coronavirus
139. We Don’t Really Know How Many People Have Coronavirus
140. We Knew Disease X Was Coming. It’s Here Now.
141. We Must Vote in November. This Is How to Ensure That We Can.
142. We Need Amazon During the Coronavirus. That’s a Problem.
143. We’re Doing What We Can to Keep Truckers on the Road
144. We’re Reading the Coronavirus Numbers Wrong
145. We’re Relying on Trump to Care About Our Lives
146. What America Needs Next： A Biden National Unity Cabinet
147. What Cuomo Hasn’t Done
148. What Happens if America’s 2.5 Million Farmworkers Get Sick？
149. What Happens if Congress Cannot Assemble to Do Its Work？
150. What If We Have to Decide Who Gets a Ventilator？
151. What It’s Like to Run a Rural State During a Pandemic
152. What Moms Always Knew About Working From Home
153. What the Plague Can Teach Us About the Coronavirus
154. What the World Needs Now Is Grace
155. What Was the Last Time an Economy Froze Like This？
156. What You Should Know Before You Need a Ventilator
157. When a Pandemic Strikes Americans Who Are Already Suffering
158. When Coronavirus Quarantine Is Class Warfare
159. When Innovation Looks Like Dancing Dinosaurs
160. When Will There Be a Treatment for the Coronavirus？
161. Where Can Domestic Violence Victims Turn During Covid-19？
162. Where Is God in a Pandemic？
163. Why Are So Many More Men Dying from Coronavirus？
164. Why Is America Choosing Mass Unemployment？
165. Why Telling People They Don’t Need Masks Backfired
166. Why the Coronavirus Is So Much Worse Than Sept. 11
167. Why the Wealthy Fear Pandemics
168. Will Our Economy Die From Coronavirus？
169. Will the Coronavirus Threaten Our Food？
170. Will We Flunk Pandemic Economics？
171. Will We Have an America Without Restaurants？
172. With Coronavirus, ‘Health Care for Some’ Is a Recipe for Disaster
173. Would Mozart Have Performed for You on Zoom？
174. Your Building Can Make You Sick or Keep You Well
175. Your Friendly Tech Bro Might Be Looking for a Loan
